# Ubiquitin‐specific protease 2 regulates Ang Ⅱ–induced cardiac fibroblasts activation by up‐regulating cyclin D1 and stabilizing β‐catenin in vitro

**DOI:** 10.1111/jcmm.16162

**Published:** 2020-12-12

**Authors:** Qiong Xu, Mingke Liu, Fangcheng Zhang, Xiaolin Liu, Sisi Ling, Xuke Chen, Jielei Gu, Wenchao Ou, Shiming Liu, Ningning Liu

**Affiliations:** ^1^ Guangzhou Institute of Cardiovascular Disease Guangdong Key Laboratory of Vascular Diseases State Key Laboratory of Respiratory Disease The Second Affiliated Hospital, Guangzhou Medical University Guangzhou China

**Keywords:** cardiac fibrosis, collagen, proliferation, USP2, β‐catenin

## Abstract

Cardiac fibrosis, featuring abnormally elevated extracellular matrix accumulation, decreases tissue compliance, impairs cardiac function and accelerates heart failure. Mounting evidence suggests that the ubiquitin proteasome pathway is involved in cardiac fibrosis. In the present study, ubiquitin‐specific protease 2 (USP2) was identified as a novel therapeutic target in cardiac fibrosis. Indeed, USP2 expression was increased in angiotensin II–induced primary cardiac fibroblasts (CFs) from neonatal rats. In addition, USP2 inhibition suppressed CFs proliferation, collagen synthesis and cell cycle progression. Furthermore, USP2 interacted with β‐catenin, thereby regulating its deubiquitination and stabilization in CFs. To sum up, these findings revealed that USP2 has a therapeutic potential for the treatment of cardiac fibrosis.

## 1 INTRODUCTION

Cardiac fibrosis features an imbalance between the production and degradation of the extracellular matrix (ECM), leading to excessive ECM accumulation and promoting the progression of cardiac dysfunction.^1^, ^2^ In addition, excessive ECM deposition not only causes ventricular dilation, cardiac remodelling and infarct expansion, but also leads to changes in myocardial structure, phenotype and function.^3^ The ECM comprises collagens, elastic fibres, glycosaminoglycan and glycoproteins, mostly deriving from fibroblasts.^4^ Proliferation of cardiac fibroblasts (CFs) and increased accumulation of collagen in the cardiac interstitium is the leading cause and main hallmark of cardiac fibrosis.^5^ Wnt signalling represents one of the major molecular pathways regulating the cell fate in the animal kingdom.^6^ Moreover, it profoundly affects developmental processes during embryogenesis and has critical functions in tissue homeostasis, cell renewal and regeneration in adults.^6^, ^7^ Wnt ligands are highly evolutionarily conserved glycoproteins, which upon interaction with receptor complexes initiate several intracellular signalling cascades including canonical and non‐canonical networks. In canonical pathways, Wnt ligands interact with frizzled/low‐density lipoprotein receptor‐related proteins receptor complexes to induce multiple molecular reactions causing cytoplasmic stabilization of β‐catenin, which undergoes nuclear translocation and up‐regulates Wnt‐specific genes controlling the cell fate in multiple cells and tissues at the transcriptional level.^8^, ^9^ Notably, mounting evidence suggests that induced canonical Wnt signalling might play a critical role in fibrogenesis. Currently, pathological activation of the canonical Wnt pathway is considered to be involved in the pathogenetic mechanisms of pulmonary, dermal, renal, hepatic and cardiac fibrosis.^10^, ^11^ Recent research has demonstrated Wnt pathway up‐regulation after myocardial infarction in progenitor cells, endothelial cells, leucocytes and fibroblasts in the whole myocardium, indicating widespread functions for the Wnt pathway in cardiac repair.^12^

The ubiquitin proteasome system (UPS), which is composed of ubiquitin‐conjugating complexes, deubiquitinating enzymes (DUBs) and the proteasome, highly affects the dynamic balance between protein synthesis and degradation.^13^, ^14^ Mounting evidence suggests that the UPS is critical for the pathogenesis of multiple cardiovascular diseases.^15^ As a DUB, USP2 can reverse target protein degradation via removal of the ubiquitin/ubiquitin chain from the respective substrates and regulate many cell processes such as DNA damage responses, cell cycle regulation and many signalling pathways.^16^ Previous studies have identified two isoforms of USP2, which exists as two isoforms of 45 and 69 kDa, namely USP2‐45 and USP2‐69, or USP2a and USP2b.^17^, ^18^ ML364 represents a well‐characterized non‐covalent USP2 suppressor and is considered a critical probe of USP2 biology. A study showed that ML364 directly targets cyclin D1 for degradation through USP2 suppression, resulting in inhibited colorectal and mantle cell lymphoma cell lines.^19^ In addition, USP2 promotes the nuclear accumulation and transcriptional activity of β‐catenin, resulting in elevated expression of Wnt/β‐catenin pathways’ target genes. What's more, either genetic knockdown or pharmacological inhibition of USP2 leads to β‐catenin destabilization.^20^ Moreover, USP2 is overexpressed in multiple malignancies such as glioma,^21^ prostate cancer^17^ and bladder cancer,^22^ indicating it might contribute to cancer cell proliferation and metastasis. Nevertheless, USP2’s association with cardiovascular disease is undefined and deserves further investigation. In this study, a cellular model of angiotensin II(Ang II)–induced cardiac fibroblasts activation was established to investigate USP2’s effects on cardiac fibrosis and explore the underlying mechanisms. In addition, the potential relationship between USP2 and β‐catenin in CFs was determined.

## 2 METHODS

### 2.1 Materials

ML364 was manufactured by MedChemExpress (USA). Ang II was provided by Sigma‐Aldrich (USA). MG132 was a product of Enzo (USA). Anti‐USP2 and anti‐periostin antibodies were purchased from Proteintech (China). Anti‐collagen‐III antibody was manufactured by Abcam (United Kingdom). Anti‐CTGF and anti‐GAPDH antibodies were obtained from Boster (China) and Bioworld (Bioworld Technology, USA), respectively. Antibodies raised against ubiquitin, cyclin D1, P27, β‐catenin, GSK‐3β and P‐ GSK‐3β(Ser9) were provided by Cell Signaling Technology (USA). MTS assay reagents were manufactured by Promega (USA). The Co‐IP assay kit was purchased from Life Technologies (USA). Cell Cycle Detection Kit was manufactured by Keygen (China). The enhanced chemiluminescence (ECL) kit was provided by Santa Cruz Biotechnology (USA). Antibodies were diluted at 1:1000, 1:500 and 1:50 for immunoblot, immunofluorescence and Co‐IP assay, respectively.

### 2.2 Culture and treatment of cardiac fibroblasts

One‐to‐two‐day‐old SD rats were provided by Guangdong Medical Laboratory Animal Center (China). CFs from neonatal rats were isolated as described previously.^23^ Briefly, upon dissociation from the cardiac tissue with trypsin and collagenase Type II (Gibco), cell culture was performed for 2 hours at 37°C, and non‐adherent cells were removed. CFs were routinely maintained in DMEM (Gibco) containing 10% FBS and penicillin (100 U/mL)‐streptomycin (100 µg/mL) at 37°C in a humid environment containing 5% CO_2_. CFs were subcultured at 90% confluence, and cells at passages 2 to 4 were used in subsequent experiments.

When cell confluence reached 30%–40%, the culture medium was replaced with serum‐free DMEM, and the cells underwent treatment with Ang II (2 µmol/L) and/or ML364 (0.5 µmol/L), for 72 hours.

### 2.3 Cell viability assay

Cell viability was assessed by the MTS assay as reported previously.^24^ In brief, 100 μL cell suspensions were seeded in 96‐well plates; after treatment, 20 µL MTS solution was added per well, and optical density was obtained at 490 nm.

### 2.4 Immunoblot

Immunoblot was carried out as described in our previous report.^25^, ^26^ Briefly, equal amounts of protein were electrophoretically resolved and electro‐transferred onto polyvinylidene difluoride (PVDF) membranes, which were probed with primary antibodies targeted to various proteins. This was followed by incubation with adequate secondary antibodies. For semiquantitative detection of immunoreactive bands, the samples were exposed to X‐ray films (Kodak, Japan) after incubation with ECL reagents.

### 2.5 5‐ethynyl‐2 0‐deoxyuridine (EdU) proliferation assay

This assay was carried out as previously described.^27^ Briefly, cells were treated and further incubated in growth medium with EdU (100 μmol/L) for further 2 hours and fixed by addition of 4% paraformaldehyde for 10 minutes. After cell permeabilization with 0.2% Triton X‐100 (10 minutes), incubation was performed with reaction buffer for 30 minutes in the dark. This was followed by Hoechst (5 mg/mL) staining for 30 minutes and imaging under a fluorescence microscope. EdU‐positive cells were counted, and data were presented as EdU‐stained/Hoechst‐positive cells × 100 (%).

### 2.6 Cell cycle assay

CFs were cultured in serum‐free DMEM for 24 hours before treatment. Then, they were collected, washed with cold PBS thrice, and incubated with 500 μL PBS and 2 mL 70% alcohol at 4°C overnight. After two washes with cold PBS, the samples were incubated with PI and RNase A for 30 minutes at 4°C away from light. The samples were finally assessed flow cytometrically.

### 2.7 Immunohistochemical staining

CFs were seeded into 24‐well plates, treated and fixed with chilled 4% formalin for 15 minutes. After permeabilization with 0.1% Triton X‐100 (10 minutes), blocking was carried out with 5% BSA for 30 minutes. This was followed by successive incubations with primary (in 1% BSA at 4°C overnight) and secondary (at ambient for 1 hour) antibodies. Finally, DAPI was used to stain the nuclei. Images were captured by fluorescence microscopy.

### 2.8 Co‐immunoprecipitation (Co‐IP)

The Co‐IP procedure has been described previously.^28^, ^29^ Briefly, Dynabeads were linked to antibodies for 16–24 hours, followed by addition of CF lysates for 2 hours (4°C). Immuno‐complexes underwent resuspension with SDS blue loading buffer and separation from the Dynabeads. The proteins were subjected to SDS‐PAGE and immunoblot.

### 2.9 Plasmid transfection

For silencing USP2, shRNA plasmids were transfected into CFs with the Lipofectamine 3000 (Invitrogen), according to the standard protocol. The designed shRNA and NC‐shRNA were obtained from Genechem Co., Ltd. (Shanghai, China). Target sequences of shRNA were 5′‐CAGGAATTCCTCCGTTTCCTT‐3′. Transfected CFs were treated with AngII (2 μmol/L) after 6h and cultured for 48 or 72 hours before further analyses.

### 2.10 Wound healing assay

Cells were seeded into 6‐well dishes and cultured to confluence. A sterilized 100 μL pipette tip was used to generate a scratch through the diameter, and the debris was washed away. The wound area was captured by inverted microscope before and 48 h after the treatment. Then, width of the wound area was evaluated using ImageJ software.

### 2.11 Statistical analyses

Data are mean ± SD. Group differences were assessed by one‐way analysis of variance (ANOVA) or *t* test, as appropriate. Statistical analysis was performed with SPSS (SPSS, USA), with *P* < 0.05 indicating statistical significance.

## 3 RESULTS

### 3.1 USP2 expression is increased in activated cardiac fibroblasts

**FIGURE 1:**
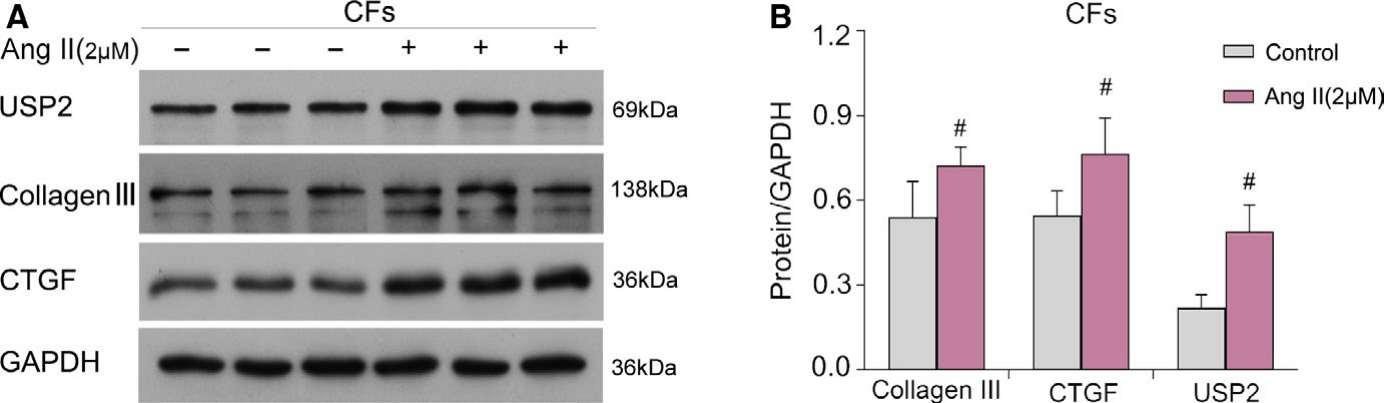
USP2 expression in activated cardiac fibroblasts. A, B, CFs were administered Ang II for 72 h, and protein amounts of collagen Ⅲ, CTGF, and USP2 were determined by immunoblot. GAPDH was employed for normalization. ^#^ *P* < 0.05 vs control group. Mean ± SD (*n* = 3)

To examine the potential involvement of USP2 in cardiac fibrosis *in vitro*, a cellular model of cardiac fibrosis was established as described above. Immunoblot was performed for evaluating marker proteins of cardiac fibroblast activation, including collagen‐III and connective tissue growth factor (CTGF), as well as USP2 in Ang II–induced CFs. As shown in Figure 1, collagen‐III, CTGF and USP2 protein expression levels were significantly increased under Ang II stimulation. The above findings suggested USP2 to be a critical positive modulator of Ang II–induced cardiac fibrosis in vitro.

### 3.2 USP2 regulates Ang II–induced cell proliferation and migration

**FIGURE 2:**
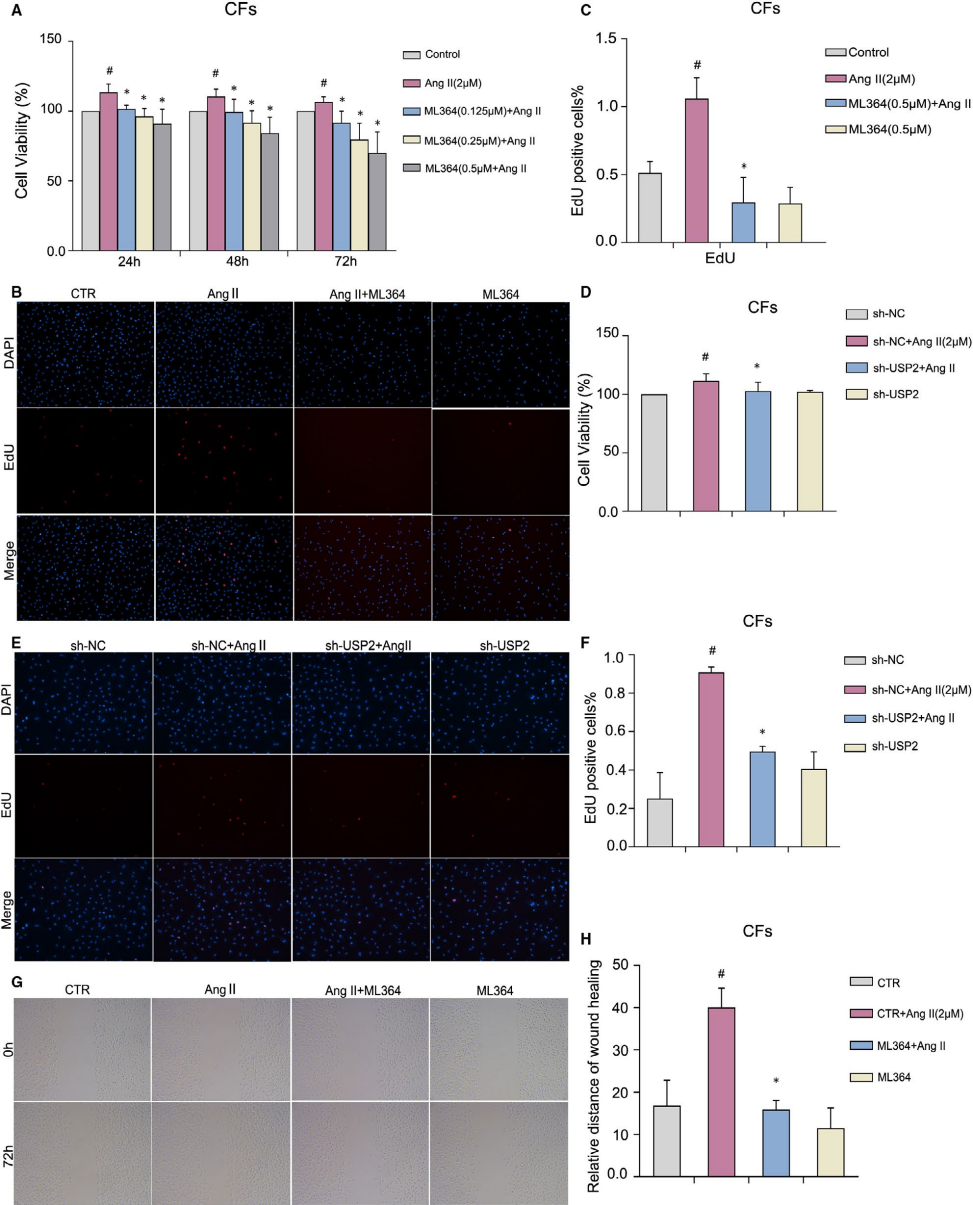
USP2 regulates Ang II‐induced cell proliferation and migration in CFs. A, To assess the inhibitory effect of ML364, CFs were treated with Ang II (2 μmol/L) or different concentrations (0.15, 0.25, or 0.5 μmol/L) of ML364 for 24, 48 and 72 h, respectively, and evaluated by the MTS assay. B, C, DNA synthesis in ML364 (0.5 μmol/L) treated CFs was examined by the EdU assay under Ang II (2 μmol/L) stimulation for 72 h. scale bar: 50 μm. D, Transfected CFs were treated with Ang II (2 μmol/L) after 6 h and cultured until 72 h, then evaluated by the MTS assay. E, F, DNA synthesis in transfected CFs was examined by the EdU assay for 72 h. scale bar: 50 μm. G, H, Wound healing assay was used to detect cell migration ability after ML364 treatment. ^#^ *P* < 0.05 vs control group, *P < 0.05 vs Ang II treatment group

Overproliferation of cardiac fibroblasts is an important event in cardiac fibrosis, and its inhibition is expected to alleviate fibrosis. To determine whether USP2 inhibition may reverse the effects of Ang II–induced proliferation, CFs were administered various ML364 amounts for distinct times, followed by the MTS assay. There was no obvious cytotoxic effect of ML364 at any concentration (Figure 2A). In addition, ML364 at 0.5 μmol/L reduced cell viability significantly but did not exert cytotoxic effects. Therefore, 0.5 μmol/L of ML364 was used in subsequent experiments. Cell transfection results also showed that silencing USP2 inhibited the cell viability of CFs (Figure 2D). On the other hand, the proliferation of CFs was also detected by EdU staining. Fluorescence microscopy following EdU staining revealed an overt reduction in EdU‐positive cells in ML364 treatment groups (Figure 2B,C). The same results were obtained in the sh‐USP2 treatment group (Figure 2E,F). The effect of ML364 on CFs migration was evaluated by wound healing assay. The results showed that inhibition of USP2 hampered CFs migration (Figure 2G,H).

### 3.3 ML364 inhibits cell cycle progression

**FIGURE 3:**
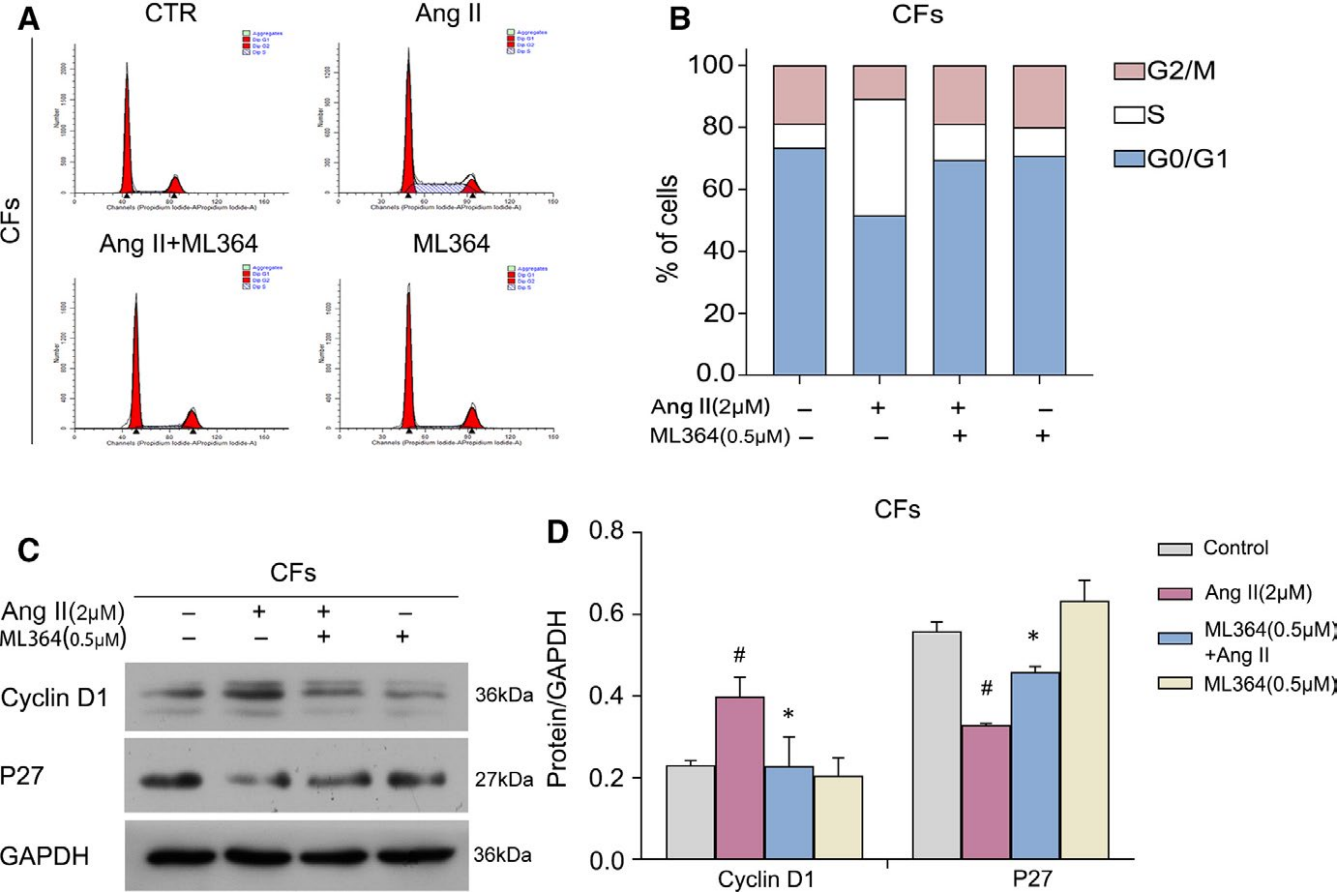
USP2 inhibition causes cell cycle arrest in activated cardiac fibroblasts. A, B, CFs were grown under starvation conditions for 24 h before treatment with ML364 (0.5 μmol/L) and Ang II (2 μmol/L) in serum‐free medium for an additional 24 h. Representative graphs are shown. Cell cycle distribution was then assessed. Mean ± SD (*n* = 3). C, D, Protein lysates were obtained from CFs treated with Ang II (2 μmol/L) or ML364 (0.5 μmol/L) for 72 h. Cyclin D1 and p27 proteins were detected by immunoblot, with GAPDH employed for normalization. ^#^ *P* < 0.05 vs control group, *P < 0.05 vs Ang II treatment group

The experiments described above investigated the antiproliferative effect of ML364. Given that DUBs have a vital function in deciding the fate of proteins that control the cell cycle,^30^ we sought to further explore the mechanisms by which ML364 inhibits cell growth. Cell cycle progression was evaluated flow cytometrically. Notably, ML364 arrested the cell cycle at the G0/G1 phase (Figure 3A,B). Then, Western blot was performed to detect the levels of proteins associated with cell cycle regulation. The results demonstrated that ML364 decreased the expression levels of cyclin D1 in CFs, while p27 protein expression was increased in CFs (Figure 3C,D). The above findings indicated that ML364 induced G0/G1 cell cycle arrest by modulating cyclin D1 and p27.

### 3.4 USP2 regulates Ang II–induced cardiac fibroblasts activation

**FIGURE 4:**
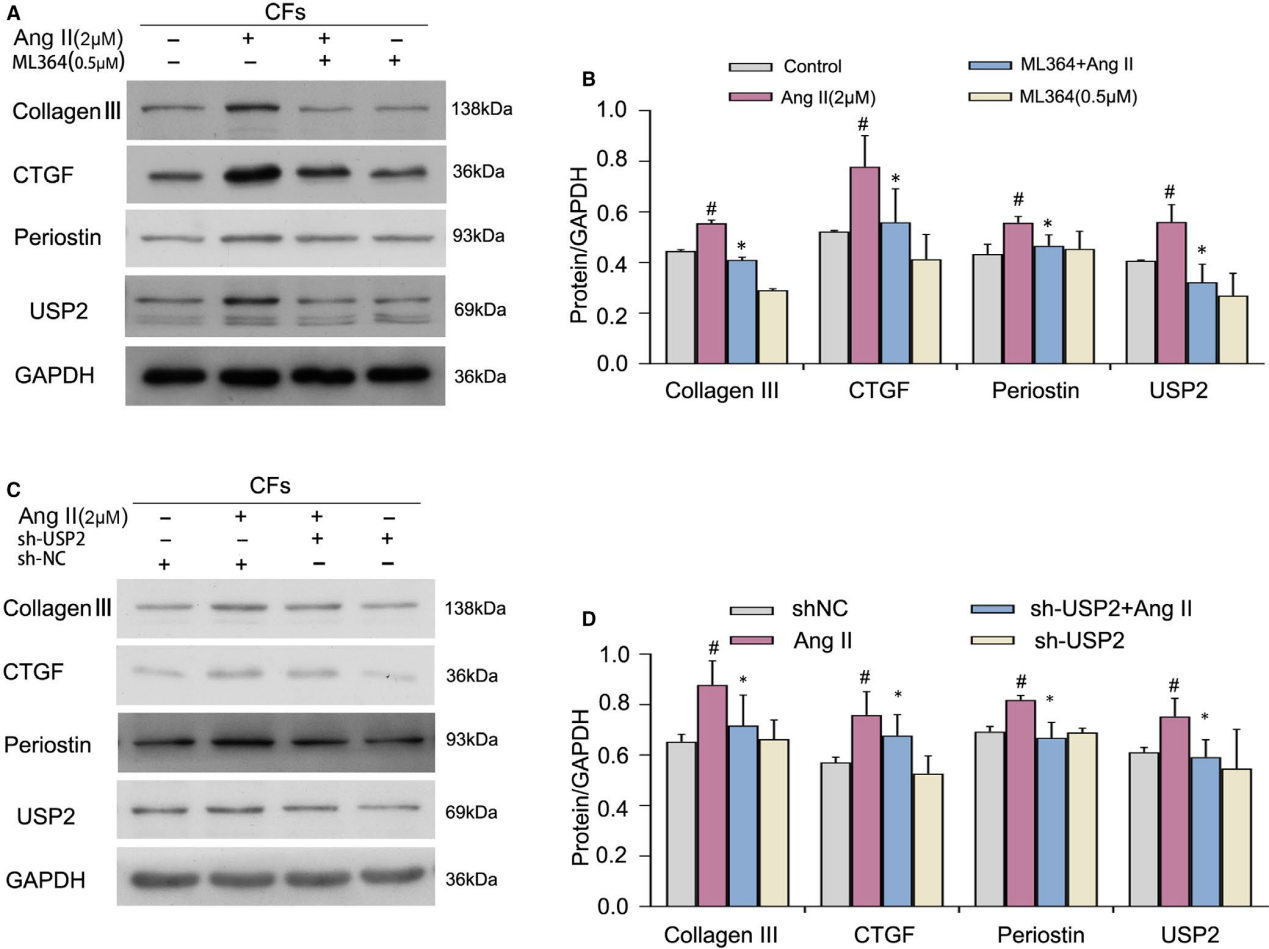
The effect of ML364 treatment on Ang II–induced changes in USP2, collagen‐III, periostin and CTGF protein expression in cultured cardiac fibroblasts. A, C, CFs underwent treatment with Ang II (2 μmol/L) or ML364 (0.5 μmol/L) for 72 h or cells were treated with USP2 shRNA for 48 h, and protein amounts of collagen Ⅲ, CTGF, Periostin and USP2 were evaluated by immunoblot. GAPDH was employed for normalization. Statistical charts are shown in (B, D). ^#^ *P* < 0.05 vs control group, *P < 0.05 vs Ang II treatment group

Cardiac fibrosis could also be monitored by assessing collagen production and quantitating other fibrosis‐associated proteins. As shown by Western blot analysis (Figure 4A‐D), Ang II overtly increased USP2 protein levels in cardiac fibroblasts, which were suppressed by ML364. Fibrosis‐associated markers, including collagen‐III, Periostin and CTGF, were all markedly up‐regulated by Ang II but inhibited by ML364 and sh‐USP2 treatment. Above results indicate that inhibition of USP2 can prevent the activation of cardiac fibroblasts, thus avoiding the further development of cardiac fibrosis.

### 3.5 USP2 inhibition inactivates cardiac fibroblasts in vitro via the Wnt/β‐catenin pathway

**FIGURE 5:**
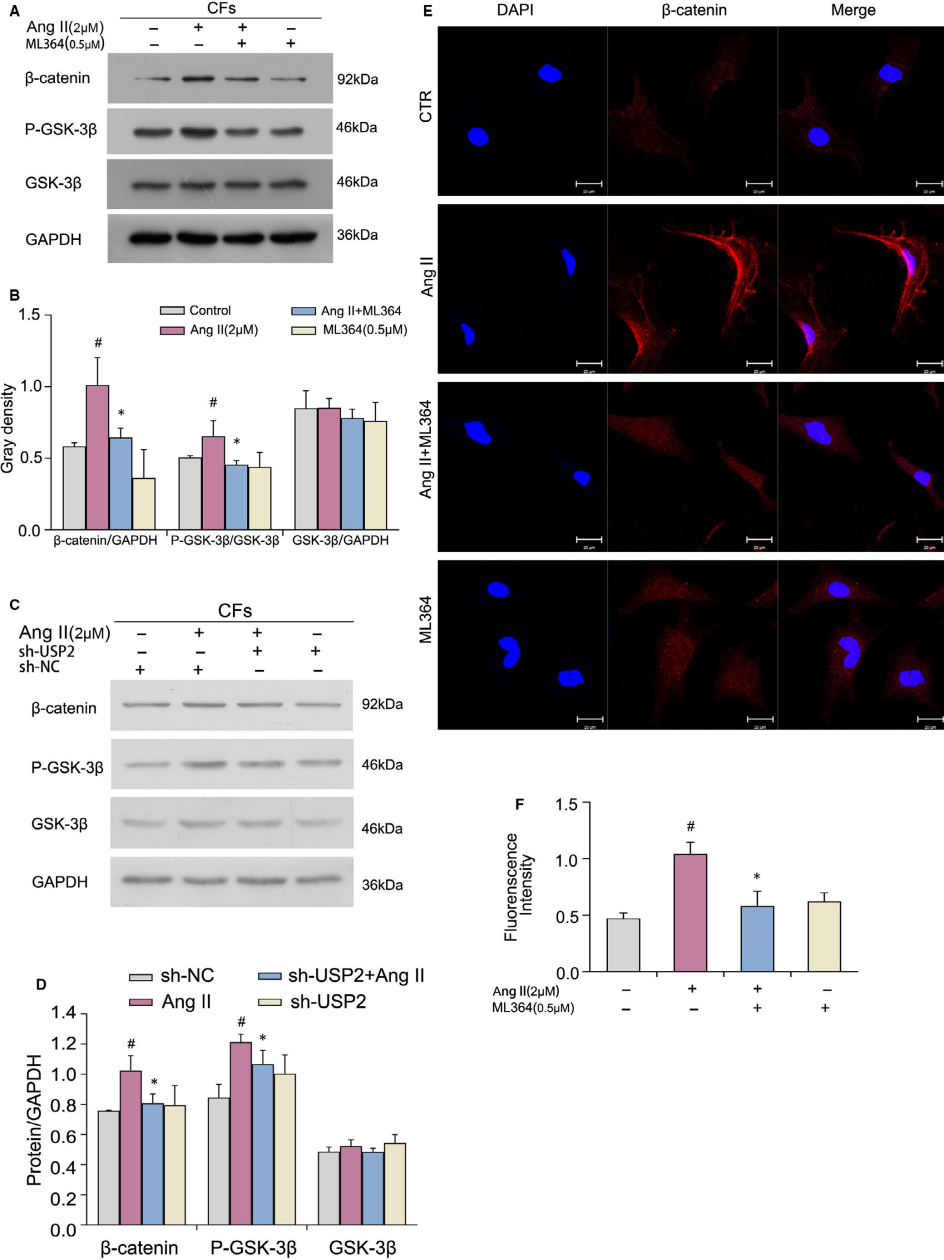
Effects of USP2 on Ang II–induced Wnt/β‐catenin pathway activation in cardiac fibroblasts. A‐D, CFs were incubated with Ang II (2 μmol/L) and/or ML364 (0.5 μmol/L) for 72 h or cells were treated with USP2 shRNA for 48 h. Then, β‐catenin, P‐GSK‐3β and GSK‐3β protein levels were assessed by immunoblot. GAPDH was employed for normalization. ^#^ *P* < 0.05 vs control group, *P < 0.05 vs Ang II treatment group. Mean ± SD (*n* = 3). E, F, CFs were treated as in (A and B). Representative images and quantitated fluorescence intensities are shown. ^#^ *P* < 0.05 vs control group, *P < 0.05 vs Ang II treatment group. Mean ± SD (*n* = 3)

Wnt*/β*‐catenin signalling is critical for the development and progression of various fibrotic diseases. Western blot was carried out to detect the levels of proteins related to Wnt/*β*‐catenin signalling such as β‐catenin, GSK‐3β and p‐GSK‐3β. As shown in Figure 5A‐D, the Wnt/β‐catenin pathway was activated in CFs after treatment with Ang II, which promoted the phosphorylation of GSK‐3β and increased the protein levels of β‐catenin significantly. However, ML364 could reverse these effects, inactivating Wnt/*β*‐catenin signalling, which is the same as the result of silence USP2. Besides, immunofluorescent staining was performed to determine whether USP2 inhibition affects Wnt/β‐catenin pathway activation. Consistent with the WB results, ML364 could reverse the effects of Ang II (Figure 5E,F). To further detect the interaction between β‐catenin and USP2, Co‐IP was performed.

### 3.6 USP2 inhibition inactivates cardiac fibroblasts in vitro by deubiquitinating and stabilizing β‐catenin

**FIGURE 6:**
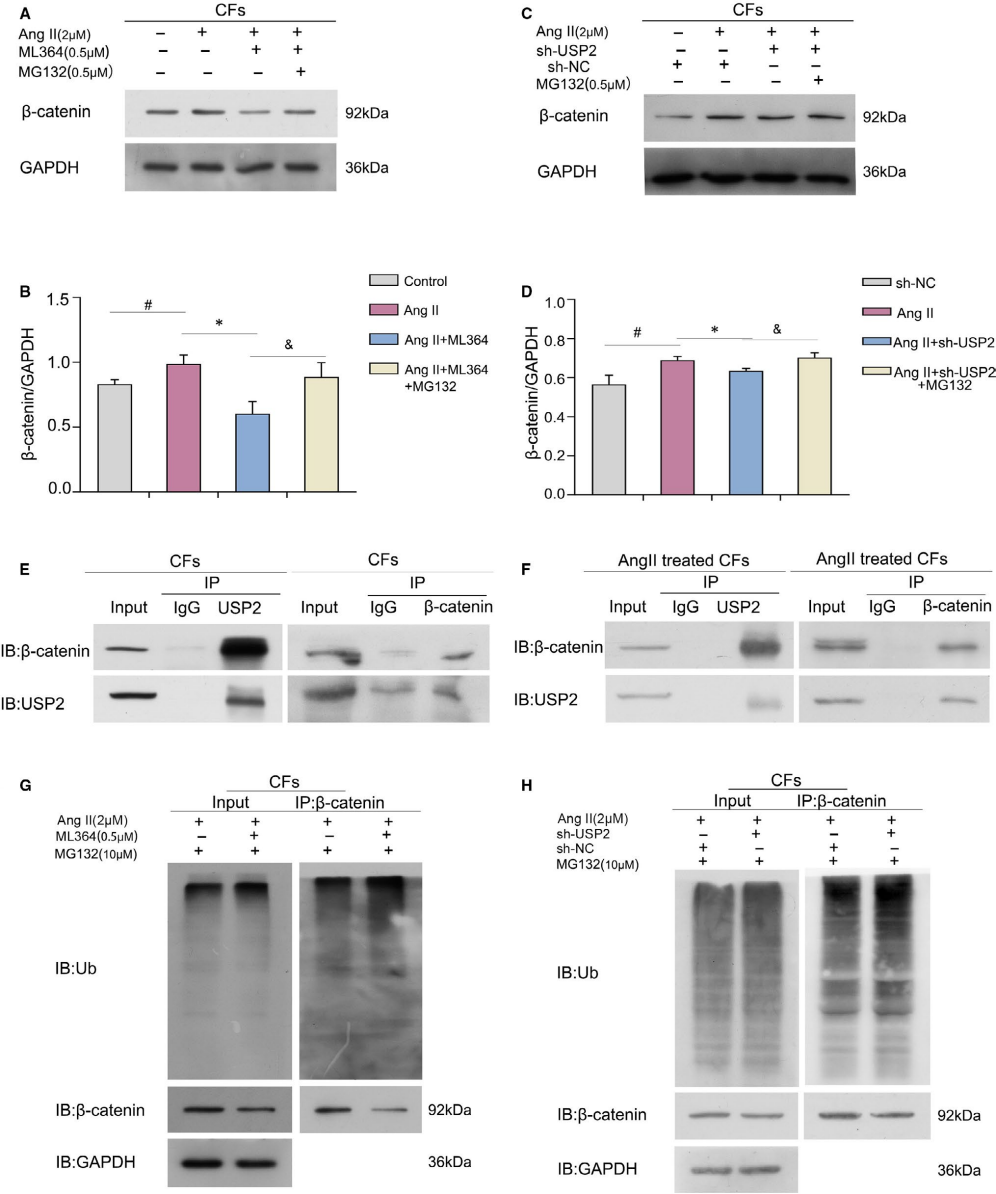
USP2 regulates Ang II–induced cardiac fibroblasts activation by deubiquitinating and stabilizing β‐catenin. (A and B) CFs were incubated with Ang II (2 μmol/L), ML364 (0.5 μmol/L) and MG132 (0.25 μmol/L), respectively, for 72 h. C, D, Transfected CFs were treated with Ang II (2 μmol/L) and MG132 (0.25 μmol/L) after 24 h and cultured another 24 h. Cell lysates were prepared and Western blot analysis was performed for determining β‐catenin expression. ^#^ *P* < 0.05 vs control group, *P < 0.05 vs Ang II treatment group, ^&^ *P* < 0.05 vs Ang II+ML364(sh‐USP2)‐treatment group. Mean ± SD (*n* = 3). E, F, Cell extracts from CFs that with or without Ang II treatment were submitted to co‐immunoprecipitation. Immunoblot was carried out to assess USP2’s interaction with β‐catenin. G, H, Cells were incubated with Ang II (2 μmol/L) and/or ML364 (0.5 μmol/L) for 72 h while transfected CFs were cultured for 48 h, and MG132 (10 μmol/L) for additional 6 h. Then, co‐immunoprecipitation was carried out with β‐catenin followed by immunoblot with antibodies targeting ubiquitin (Ub) and β‐catenin

We hypothesized that β‐catenin down‐regulation at the protein level occurs through ubiquitin proteasome system–mediated protein degradation. As depicted in Figure 6A‐D, MG132 (20S proteasome inhibitor) efficiently reversed β‐catenin down‐regulation associated with USP2 inhibition, suggesting that reduced β‐catenin resulted from proteasome‐associated protein degradation. Hence, we proposed that inhibition of USP2‐induced β‐catenin down‐regulation occurred through β‐catenin degradation. To investigate the potential interaction between β‐catenin and USP2, co‐immunoprecipitation (Co‐IP) was performed. As expected, we found that USP2 notably interacted with β‐catenin in CFs (Figure 6E). In order to assess whether the effects of Ang II had impact on their interactions, CFs were tested again by Co‐IP after Ang II treatment, and the results showed that the interactions were continuous and stable (Figure 6F). To further confirm that USP2 regulates β‐catenin degradation through deubiquitination, Co‐IP was performed to quantitate ubiquitinated β‐catenin. Interestingly, USP2 inhibition markedly elevated the amounts of ubiquitinated β‐catenin, suggesting USP2 as a DUB for β‐catenin, capable of reversing β‐catenin ubiquitination, thereby stabilizing the β‐catenin protein (Figure 6G,H).

## 4 DISCUSSION

Cardiovascular disease represents the main cause of death worldwide. It is aggravated by cardiac fibrosis, characterized by excessively high ECM accumulation as a dynamic remodelling process that the heart undergoes during ischaemic injury or stress overload; cardiac fibrosis involves multiple cell types such as cardiomyocytes (CMs), endothelial cells, immune cells and CFs.^31^ However, the development of antifibrotic therapeutics for cardiovascular disease is seriously hampered. For example, the adult heart has limited regenerative potential, and cardiomyocytes are incapable of proliferating to a degree allowing the replacement of the injured myocardium.^32^ This limit treatments aiming to suppress fibrosis completely, as endogenous CMs cannot replace the lost muscle tissue, thereby promoting cardiac rupture. Cardiac fibroblasts, constituting the largest cell population of the heart, are key components of cardiac fibrosis due to their ability to secrete and degrade the ECM. Therefore, it is an important strategy to assess the development of cardiac fibrosis in CFs.^33^

In the UPS, deubiquitinase (DUB) plays a fundamental role thanks to its ability to specifically deconjugate ubiquitin from target proteins, contributing to cell cycle control, DNA stabilization, chromatin modifications and multiple cellular pathways. There are approximately 100 DUBs in human cells, comprising six families based on sequences and structural differences.^34^ Recent evidence suggests that multiple DUBs regulate cardiac fibrosis. For example, A20 improves the heart function and inhibits cardiac fibrosis by suppressing transforming growth factor‐β‐induced kinase 1‐dependent signalling.^35^ In addition, CYLD mediates cardiac maladaptive remodelling and dysfunction through Nrf2 down‐regulation.^36^ Based on the important roles of DUBs, USP2 was assessed in a cellular model of cardiac fibrosis by Western blot. The present study demonstrated that USP2 was up‐regulated in cardiac fibrosis in vitro. Therefore, USP2 may represent a new target for regulating the development of cardiac fibrosis. It is known that pathological cardiac fibrosis leads to many types of heart diseases; therefore, these findings also provide novel insights into heart diseases that develop as a result of cardiac fibrosis.

Previous in vivo multimodal study has demonstrated that Wnt /β‐catenin canonical pathway is a major regulator of ventricular remodelling following myocardial infarction, and the activation of Wnt /β‐catenin canonical pathway has a negative impact on ventricular remodelling. However, inhibiting the Wnt /β‐catenin canonical pathway and down‐regulating β‐catenin can achieve the aim of improving the ventricular remodelling.^37^ Actually, one of the major mechanisms of β‐catenin regulation is ubiquitination, beside which DUBs, such as USP47, USP20, and USP2, have also been shown to deubiquitinate and stabilize β‐catenin directly.^20^, ^38^, ^39^ In this study, β‐catenin was down‐regulated by USP2 inhibition, which prompted us to assess the interaction between USP2 and β‐catenin. As expected, USP2 interacted with β‐catenin, and regulated its ubiquitination and degradation in CFs. Although the current results suggested that USP2 regulates β‐catenin deubiquitination, the possibility that USP2 also regulates other components in the Wnt pathway could not be ruled out, and the specific mechanisms of β‐catenin regulation by USP2 deserve further investigation.

The current study had multiple limitations. First, USP2 enhanced the inhibition of protein degradation in Ang II–induced cardiac fibroblasts activation, but their changes at the transcription level are unknown. Besides, studies have confirmed that USP2 specifically deubiquitinates cyclin D1 through direct interaction, to regulate the growth of cancer cells.^40^ Although this study demonstrated cyclin D1 protein up‐regulation in activated CFs, it remains unclear whether USP2 directly affects cyclin D1 to inhibit the development of cardiac fibrosis. In addition to USP2, the closely related USP8 is inhibited by ML364. Furthermore, a study assessed 102 kinases, including many that affect the cell cycle; however, ML364 bound to none of them, indicating USP2 as an important factor promoting the cell cycle arrest phenotype detected. This indicates that ML364 does not represent a general protease inhibitor although it targets distinct proteins.^19^ From another perspective, USP8 is closely related to USP2; therefore, it is of great significance to investigate whether the latter proteins play redundant roles in cardiac fibrosis.

In summary, the above findings suggested that USP2 contributes to the development of Ang II–induced cardiac fibroblasts activation in vitro, which is associated with β‐catenin deubiquitination and stabilization, and may be related to increased cyclin D1. As a result, USP2 should be considered a potential therapeutic target in cardiac fibrosis.

## CONFLICT OF INTEREST

The authors declare that they have no conflict of interest.

## AUTHOR CONTRIBUTION

**Qiong Xu:** Conceptualization (equal); Data curation (equal); Methodology (equal); Writing‐original draft (equal). **Mingke Liu:** Conceptualization (equal); Data curation (equal); Methodology (equal). **fangcheng zhang:** Conceptualization (equal); Data curation (equal); Methodology (equal). **Xiaolin Liu:** Conceptualization (equal); Data curation (equal); Methodology (equal). **Sisi Ling:** Conceptualization (equal); Data curation (equal); Methodology (equal). **Xuke Chen:** Conceptualization (equal); Data curation (equal); Methodology (equal). **Jielei Gu:** Conceptualization (equal); Data curation (equal); Methodology (equal). **Wenchao Ou:** Conceptualization (equal); Data curation (equal); Methodology (equal). **Shiming Liu:** Conceptualization (equal); Data curation (equal); Funding acquisition (equal); Methodology (equal); Supervision (equal); Writing‐original draft (equal); Writing‐review & editing (equal). **Ningning Liu:** Conceptualization (lead); Data curation (equal); Funding acquisition (equal); Methodology (equal); Supervision (equal); Writing‐original draft (equal); Writing‐review & editing (equal).

## Data Availability

The data that support the findings of this study are available from the corresponding author upon reasonable request.
